# Nano silicated-FeAl_2_O_4_ functionalized by *DL*-alaninium nitrate ionic liquid (FeAl_2_O_4_-SiO_2_@[*DL*-Ala][NO_3_]) as versatile promotor for aqua-mediated synthesis of spiro[chromenopyrazole-indene-triones and spiro[chromenopyrazole-indoline-diones

**DOI:** 10.1038/s41598-024-66750-2

**Published:** 2024-07-15

**Authors:** Fatemeh Molaei Yielzoleh, Kobra Nikoofar

**Affiliations:** Department of Organic Chemistry, Faculty of Chemistry, Alzahra University, Tehran, Iran

**Keywords:** Spinel FeAl_2_O_4_, *DL*-alanine, Ionic liquid (IL), Spiro[chromeno[2,3-*c*]pyrazole-4,2′-indene]triones, Spiro[chromeno[2,3-*c*]pyrazole-4,3′-indoline]diones, Domino reaction, Chemistry, Nanoscience and technology

## Abstract

In this work, the spinel FeAl_2_O_4_ was prepared and functionalized step-by-step with silica and alaninium nitrate ionic liquid ([*DL*-Ala][NO_3_]) to produce a bio-based multi-layered nanostructure (nano FeAl_2_O_4_-SiO_2_@[*DL*-Ala][NO_3_]). The obtained magnetized inorganic-bioorganic nanohybrid characterized by Fourier transform infrared spectroscopy (FT-IR), vibrating-sample magnetometry (VSM), field emission scanning electron microscopy (FESEM), energy-dispersive X-ray spectroscopy (EDAX), transmission electron microscopy (TEM), thermogravimetric analysis/differential scanning calorimetry (TGA/DSC), X-ray fluorescence (XRF), and X-Ray diffraction (XRD) analysis. A facile synthesis of some tricyclic dihydro-spiro[chromeno[2,3-*c*]pyrazole-4,2′-indene]triones and dihydro-spiro[chromeno[2,3-*c*]pyrazole-4,3′-indoline]diones via domino four-component one-pot reaction of various hydrazine derivatives, ethyl acetoacetate, heterocyclic 1,2-ketones (ninhydrin, isatin, 5-bromoisatin) and cyclic 1,3-diketones (dimedone and 1,3-cyclohexanedine), examined in the presence of nano FeAl_2_O_4_-SiO_2_@[*DL*-Ala][NO_3_] nanohybrid in refluxing aqueous media, successfully. The multi-aspect characteristics of the nanohybrid which consist of magnetized inorganic and bioorganic parts, could be the reason of its special catalytic efficacy. The recovery and reusability of the FeAl_2_O_4_-SiO_2_@[*DL*-Ala][NO_3_] magnetized nanoparticles (MNPs) were performed in two runs without significant activity loss.

## Introduction

In recent years, magnetized nanoparticles got attention as efficient catalysts in organic processes. magnetite (Fe_3_O_4_) and metal-impregnated magnetite catalysts utilized to promote different reactions (direct cross *β*-alkylation of primary alcohols, and various multicomponent reactions)^1^. Nano-magnetite, utilized as a versatile support for functionalization of various catalytic systems (such as metals, organocatalysts, *N*-heterocyclic carbenes, and chiral catalysts) yielded recyclable catalysts for development of sustainable methodologies in green chemistry and pharmaceutically significant reactions^2^.

Many types of magnetic nanoparticles including pure metals (Fe, Co, and Ni), alloys (CoPt_3_, FePt, and FeCo), iron oxides (FeO, Fe_2_O_3_, and Fe_3_O_4_) catalyzed different organic transformations^3^.

Magnetic spinel ferrites nanoparticles (MFe_2_O_4_, where M dedicated M^2+^ oxidation state of metals such as Cu, Co, Ni, Zn, Mn, and etc.) are type of ceramic oxide with magnetoelectric properties. They are known as versatile catalysts in organic reactions and transformants such as dehydrogenation, oxidation, alkylation, couplings, and etc. In fact, MFe_2_O_4_ is a Lewis acid capable of accepting electrons to form covalent bonds, which activates specific groups of reactants by increasing their electrophilicity^4–6^. The magnetites and ferrites possess special characteristics such as selectivity, stability, recyclability (due to their magnetic behavior, they could be easily separated from the reaction mixture by an external magnet and reused), reactivity, and easy preparation procedures.

FeAl_2_O_4_ is aluminum-iron single-phase spinel, in which Fe^2+^ cations occupy one eighth of the tetrahedral sites and Al^3+^ occupy one half of the octahedral sites. The distribution of those ions in the unit cell depends on the synthesis conditions, as some Fe^2+^ cations can also occupy octahedral sites^7^. FeAl_2_O_4_, which named also as iron aluminate and hercynite, rarely found in nature. It has been synthesized via various methods such as combustion reaction^7,8^, furnace heating of iron and aluminum acetylacetonate complexes^9^, microwave magnetic field (H-field) irradiation^10^, thermal treatment of mechanochemically activated (Al + Fe_3_O_4_) mixtures^11^, and pulsed laser ablation in liquid media technique^12^.

Ionic liquids (ILs) are salts that usually exist in liquid form (low-melting salts with melting point below 100 °C) and consist of organic cations and organic/inorganic anions. They also nominated as molten salts, fused salts, liquids organic salts, and liquid electrolytes^13^. ILs contain special and great properties such as wide range thermal stability, good solvating ability, high ionic conductivity, negligible vapor pressure or non-volatility at room temperature, non-flammability, low toxicity, adjustable solubility, and very low corrosivity^14,15^.

ILs are novel solvents which are "green" and "environmentally friendly" alternative of some traditional volatile organic solvents. ILs are suitable solvent replacement in different organic reactions, electrochemistry (batteries, sensors, solar cells, and separation process), industrial processes (thermal fluids and acid scavenging), spectroscopy, and material science^16–18^. In addition to their applicability in solvent media, they could be utilized as homogenous and/or heterogenous catalysts in many organic and bioorganic transcreations^19^.

Recently designation ILs based on biomolecules and natural compounds (as cations and/or anions) got special research interest due to their special characteristics such a biodegradability (that is related to green chemistry's important rule), easily preparation techniques, good chemo and/or stereo-selectivity, and accessible natural resources. The amino acid-derived ionic liquids (AAILs), AAILs could play multiple roles as solvents and/or catalysts with high catalytic activity and also as chiral additives^20^. Polysaccharide- and lignin-based ionic liquids, demonstrated catalytic applications as well as environmental remediations^21^. Carbohydrate-based ionic liquids, displayed several applications in the fields of catalysis, biomedicine, ecology, biomass, and energy conversion^22^.

Bio-ionic liquids and also catalytic systems based on alanine (ALA) amino acid, represented special roles in various transformations. Yang group in 2008, synthesized new series of ILs ([C_n_mim][Ala]) based on alanine anion and 1-alkyl-3-methylimidazolium cation ([C_n_mim], n = 2,3,4,5,6), and studied their physicochemical properties such as molecular volume, surface tension, molar enthalpy of vaporization, and thermal expansion coefficient^23^. Gathergood et al. in 2023, gained some classes of dipeptide ILs with *L*-alanine fragments and examined their microbial toxicity screening and aerobic biodegradation testing^24^. Matavos-Aramyan research group in 2019, introduced two novel chiral ionic liquids based on the *L*-( +)-alanine [which are 1-(4-((1-carboxyethyl)carbamoyl)benzyl)pyridin-1-ium chloride and 1-(4-(((1-carboxyethyl)carbamothioyl)carbamoyl)benzyl)pyridin-1-ium chloride] and examined their antibacterial and antioxidant preparties that demonstrated that they are potent to replace many antibiotic, and potentially, anticancer compounds^25^.

Presence of chromenopyrazole moiety in heterocycles, ultimate notable characteristics that made the compounds applicable in various field of chemistry, science and technology. Choi group in 2018, reported chromenopyrazole-based bipolar blue host materials for highly efficient thermally activated delayed fluorescence organic light-emitting diodes^26^. In 2023, spirocyclization of chromenopyrazoles yielded the improved inhibitory activity against the oncogenic RNA-binding protein LIN28^27^. Some chromeno[4,3-*d*]pyrazolo[3,4-*b*]pyridin-6(3*H*)-ones consist high fluorescence quantum yields, that recommended them as luminescence or fluorescence probe^28^. Bazgir research group in 2010, prepared aqueous-mediated spiro[chromeno[2,3-*c*]pyrazole-4,3′-indoline]-2′,5(6*H*)-diones via cyclocondensation reaction of isatins, 1,3-cyclohexadiones, and 3-methyl-1-phenyl-1H-pyrazol-5-ol, in the presence of *p*-TSA under reflux conditions^29^. Mukhopadhyay and co-workers in 2017, synthesized spiro[chromeno[2,3-*c*]pyrazole-4,3′-indolin]-2′,5-diones in the presence of recyclable spinel ZnFe_2_O_4_ nanopowder via three-component reaction of substituted isatins, cyclic-1,3-diketones, and 1-phenyl pyrazolone in 80 °C water medium^30^. Mohammadi Ziarani group in 2018, obtained spiro[chromeno[2,3-*c*]pyrazole-4,3′-indoline]-2′,5(6*H*)-diones via cyclocondensation reaction of isatins, 1,3-cyclohexadiones, and pyrazolone in aqueous media using sulfonic acid-functionalized mesoporous silica (SBA-Pr-SO_3_H) in refluxing water^31^. Safaei-Ghomi et al*.* in 2016, reported domino four-component reaction of hydrazines, ethyl acetoacetate, isatins, and cyclic 1,3-diketones in the presence of Fe_3_O_4_@SiO_2_-SO_3_H or Fe_3_O_4_@SiO_2_-NH_2_ NPs (as catalyst) in EtOH at 80 °C to get spiro[chromeno[2,3-*c*]pyrazole-4,3′-indoline]-diones. Fe_3_O_4_@SiO_2_-SO_3_H NPs signified as better promoter^32^. Das group in 2015, obtained functionalized tricyclic 4-spiropyrano[2,3-*c*]pyrazoles via the domino four-component reaction of hydrazines, ethyl acetoacetate, cyclic 1,2-dicarbonlys (such as ninhydrin and isatins), and cyclic 1,3-diketones in 90 °C aqueous medium using dodecylbenzenesulphonic acid (DBSA) as a Brønsted acid-surfactant combined catalyst^33^. It must be mentioned that reports for the preparation of functionalized tricyclic 4-spiropyrano[2,3-*c*]pyrazoles via the domino four component reaction of hydrazines, ethyl acetoacetate, cyclic 1,2-dicarbonlys, and cyclic 1,3-diketones are rare^32,33^.

Recently, diverse multi-layered novel magnetized silicated nanostructures get special attention in various fields of science, technology, and catalysis^34–37^. Several kinds of silicate materials has bene utilized as linkers and/or supports due to special characteristics such as hydrophilicity (due to their surface silanol groups), biocompatibility, post-functionalization capability, agglomeration avoidance of nano-sized cores, low toxicity, easily surface modification, stability, cost-effectiveness and oxidation preventer of their inner layers^38,39^.

In recent years, several types of magnetized nanohybrids (consist of bio/organic and inorganic parts) get special attention in various fields of science and technology such as nanocarrier for targeted gene delivery into HEK-293 T cells^40^, removal of toxic pollutants from contaminated water^41^, Magnetic resonance/Raman imaging^42^, and catalysts of various transformations^43^.

**Figure 1:**
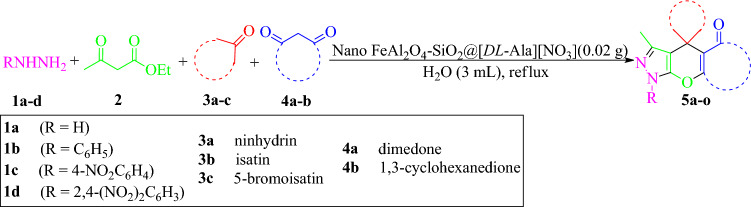
Scheme for synthesis of dihydro-1*H*-spiro[chromeno[2,3-*c*]pyrazole-4,2′-indene]-1′,3′,5(6*H*)-triones and dihydro-1*H*-spiro[chromeno[2,3-*c*]pyrazole-4,3′-indoline]-2′,5(6*H*)-diones.

In continuation of our research field in synthesizing of amino-acid based catalysts and examining their catalytic efficacy in multi-component reactions under green conditions^44–47^, herein we prepared a novel magnetized inorganic-bioorganic nanohybrid (nano FeAl_2_O_4_-SiO_2_@[*DL*-Ala][NO_3_]) via step-by-step functionalization of spinel FeAl_2_O_4_ core by silica and alaninium nitrate ionic liquid ([*DL*-Ala][NO_3_]). The alanine-based multi-layered nanostructure characterized by FT-IR, VSM, FESEM, EDAX, TEM, TGA/DSC, XRF, and XRD techniques. It catalytic efficacy examined via domino four-component one-pot reaction of various hydrazine derivatives, ethyl acetoacetate, heterocyclic 1,2-ketones and cyclic 1,3-diketones in refluxing aqueous media to obtain some dihydro-1*H*-spiro[chromeno[2,3-*c*]pyrazole-4,2′-indene]-1′,3′,5(6*H*)-triones and dihydro-1*H*-spiro[chromeno[2,3-*c*]pyrazole-4,3′-indoline]-2′,5(6*H*)-diones (Fig. 1).

## Results and discussion

### Characterization of the bio-nanostructure

**Figure 2:**
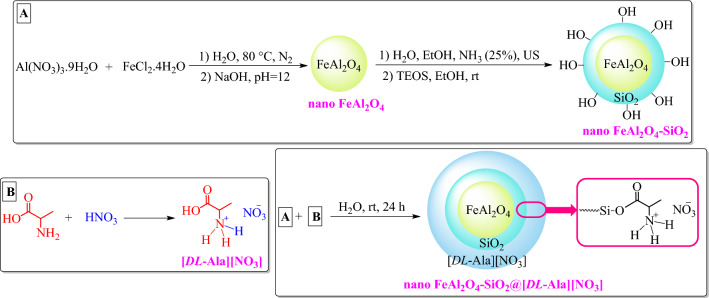
Scheme of synthetic procedure of FeAl_2_O_4_-SiO_2_@[*DL*-Ala][NO_3_].

The preparation procedure and the proposed structure of the magnetized inorganic-bioorganic nanohybrid (nano FeAl_2_O_4_-SiO2@[*DL*-Ala][NO_3_]) illustrated in Fig. 2.

**Figure 3:**
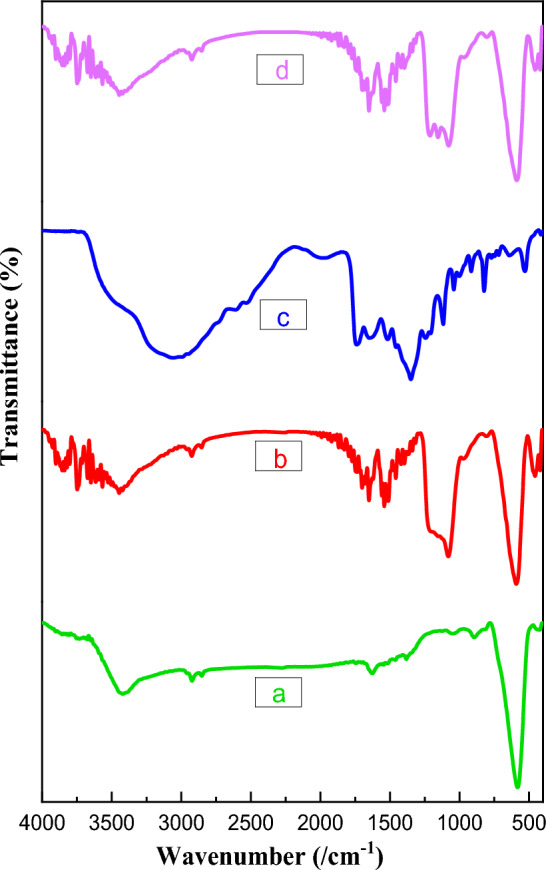
FT-IR spectra of (**a**) FeAl_2_O_4_, (**b**) FeAl_2_O_4_-SiO_2_, (**c**) [*DL*-Ala][NO_3_] IL, and (**d**) FeAl_2_O_4_-SiO_2_@[*DL*-Ala][NO_3_] MNPs.

The structure of FeAl_2_O_4_-SiO_2_@[*DL*-Ala][NO_3_] nanocomposite was studied by FT-IR spectroscopy. The FT-IR spectra of FeAl_2_O_4_ (a), FeAl_2_O_4_-SiO_2_ (b), [*DL*-Ala][NO_3_] (c), and FeAl_2_O_4_-SiO_2_@[*DL*-Ala][NO_3_] bio-nanostructure (d) demonstrated in Fig. 3. According to Fig. 3a, the peak at 441 cm^−1^, 578 cm^−1^ related to the stretching vibrations of Fe–O^48^ The peak at 1621 cm^−1^ is attributed to Al-O bond^49^ and the peak at and 3438 cm^−1^ respect to O–H bond. Based on Fig. 3b, the appearance of bands at 1076 cm^−1^ related to stretching vibration n of Si–O-Si bonds respectively. In addition, broadening the O–H starching band at 3446 cm^−1^–3741 cm^−1^, which related to Si–OH stretching vibration that combined with O–H groups of FeAl_2_O_4,_ affirmed embedding of silica on the FeAl_2_O_4_ core. According to Fig. 3c, the peaks at 1529 cm^−1^, 1346 cm^−1^, and 1140 cm^−1^ (NO_3_^−^ group)^50^, 1357 cm^−1^ (bending of methyl group), 1645 cm^−1^ and 1508 cm^−1^ (N–H bending vibrations in NH_3_^+^), and 1743 cm^−1^ (C=O stretching vibration), affirmed preparation of the [*DL*-Ala][NO_3_] ionic liquid. In addition, appearance of broad peaks in the region of 2763 cm^−1^–3317 cm^−1^ related to the O–H group in CO_2_H, which overlapped by C-H sp^3^ and NH_3_^+^ stretching vibrations in [*DL*-Ala][NO_3_]. Finally, assisting all the mentioned peaks in Fig. 3d, confirmed the synthesis of FeAl_2_O_4_-SiO_2_@[*DL*-Ala][NO_3_] bio-nanostructure.

**Figure 4:**
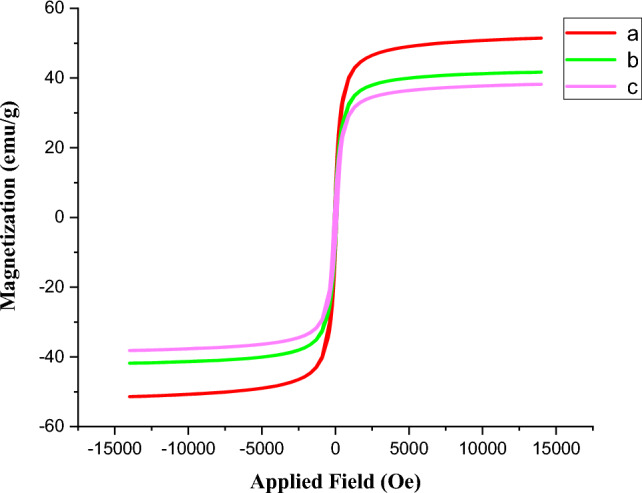
Magnetization curve of the (**a**) FeAl_2_O_4_, (**b**) FeAl_2_O_4_-SiO_2_, (**c**) FeAl_2_O_4_-SiO_2_@[*DL*-Ala][NO_3_] bio-nanostructure.

The VSM analysis of FeAl_2_O_4_, FeAl_2_O_4_-SiO_2_ and FeAl_2_O_4_-SiO_2_@[*DL*-Ala][NO_3_] nanoparticles is shown in Fig. 4. According to the curves a-c, saturation magnetization amounts of 51.436 emug^−1^ for FeAl_2_O_4_, 41.693 emug^−1^ for FeAl_2_O_4_-SiO_2_, and 38.213 emug^−1^ for FeAl_2_O_4_-SiO_2_@[*DL*-Ala][NO_3_] MNPs, confirmed the magnetic properties. Decreasing the saturation magnetization amounts form inner to outer layers, is due to the non-magnetic properties of the silica and [*DL*-Ala][NO_3_] parts, which covered the magnetized FeAl_2_O_4_ core. It must be mentioned that the saturation magnetization amounts of ~ 43 emug^−1^ was reported for FeAl_2_O_4_ previously^48^.

**Figure 5:**
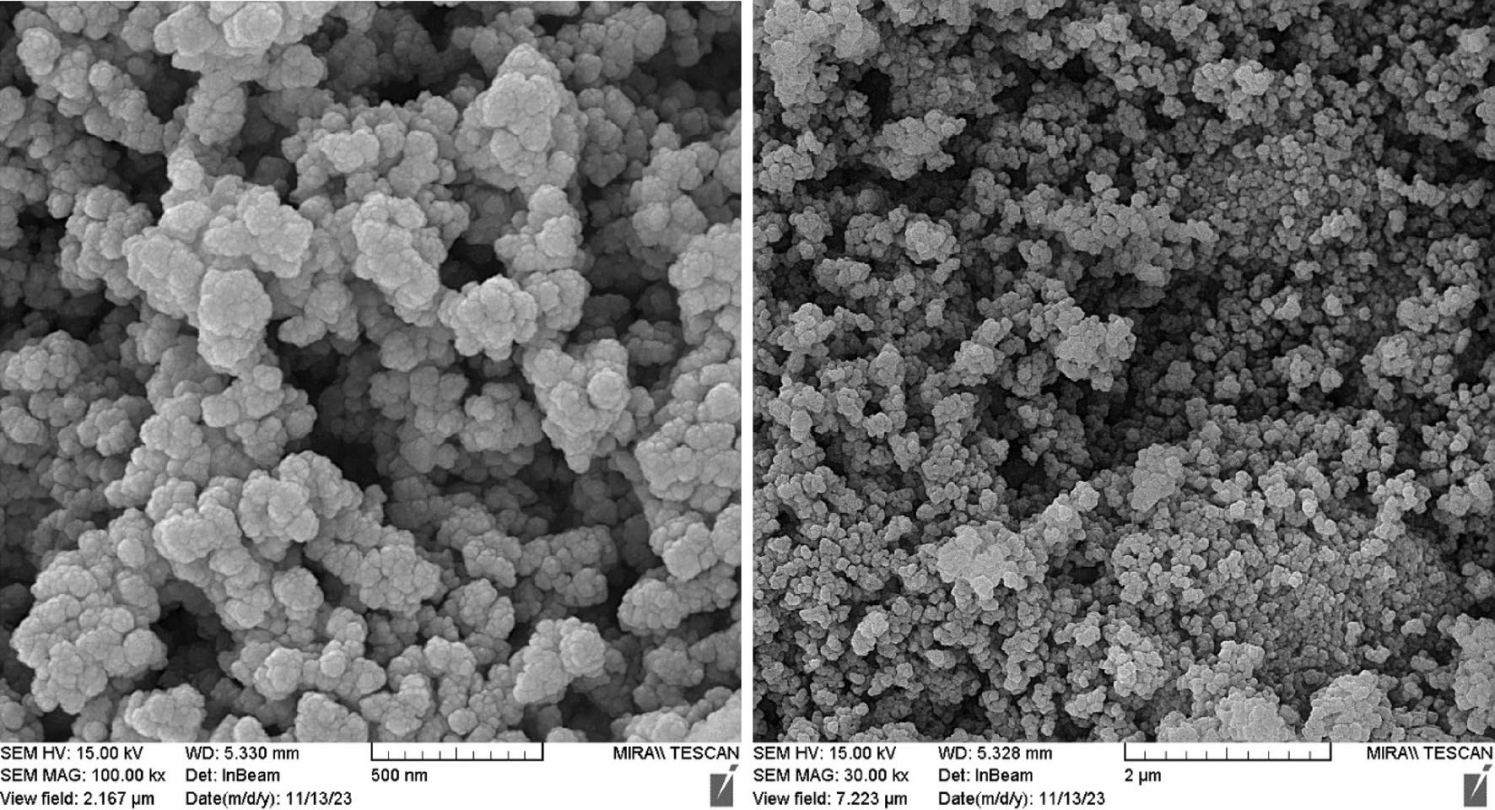
FESEM images of FeAl_2_O_4_-SiO_2_@[*DL*-Ala][NO_3_] bio-nanostructure.

The FESEM images of FeAl_2_O_4_-SiO_2_@[*DL*-Ala][NO_3_] bio-nanocomposite in 2 µm and 500 nm magnetization demonstrated in Fig. 5. The results affirmed presence of approximately agglomerated particles with semispherical shape with the average size of 55–95 nm.

**Figure 6:**
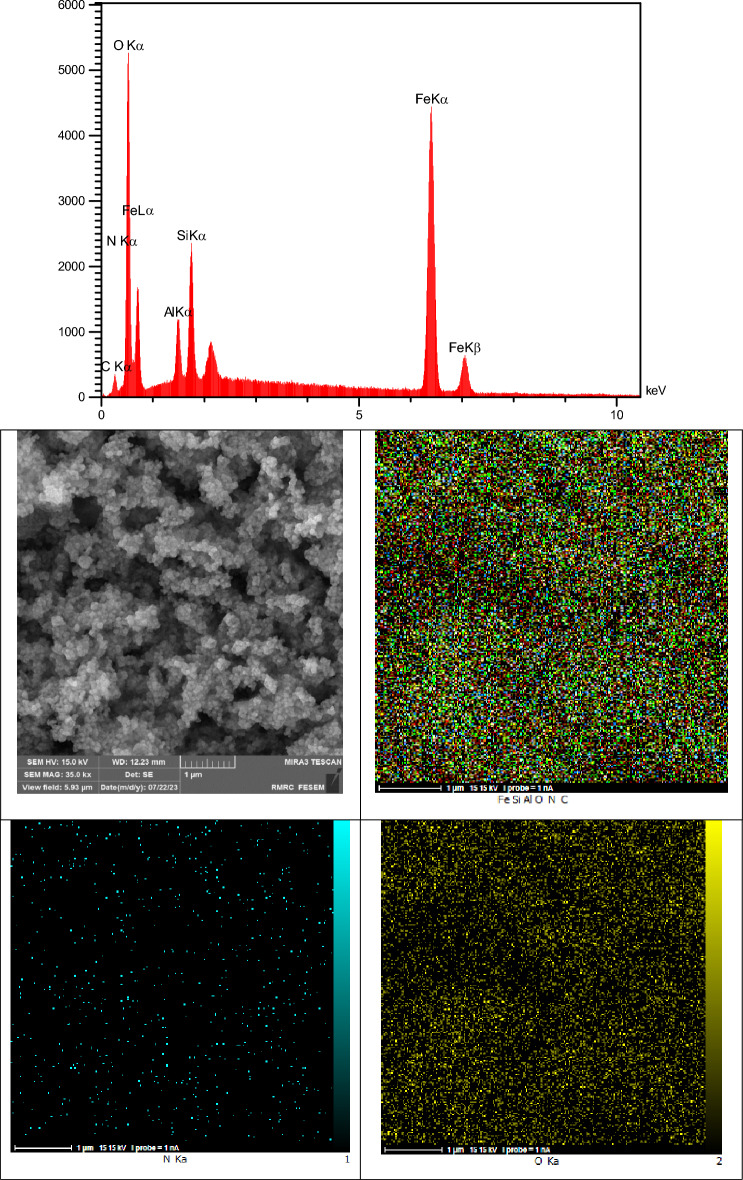

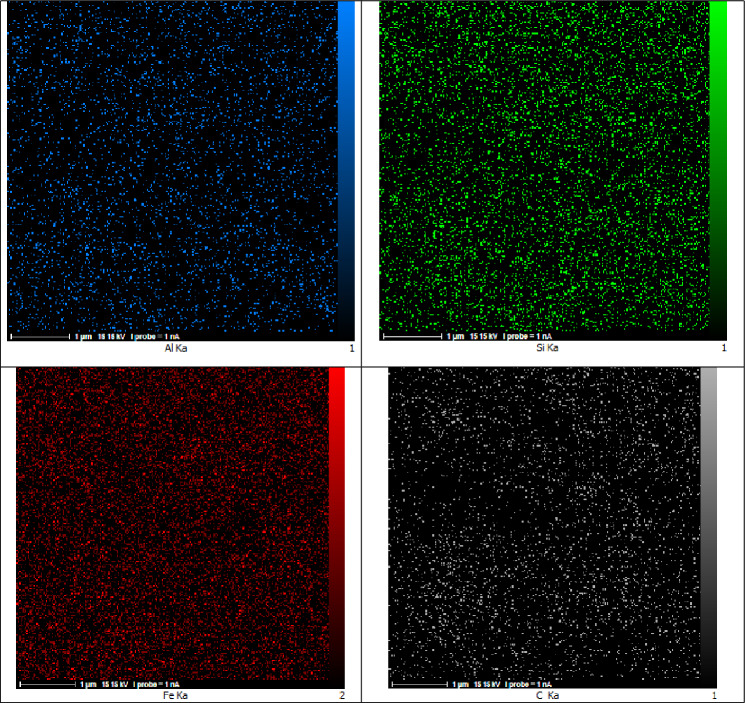
EDAX analysis and elemental mapping of nano FeAl_2_O_4_-SiO_2_@[*DL*-Ala][NO_3_].

The result of the energy-dispersive X-ray analysis and element distribution image (EDAX mapping) represented in Fig. 6. The data revealed the presence of Al (2.20 W%), Fe (54.87 W%), Si (4.36 W%), C (7.37 W%), N (1.16 W%) and O (30.06 W%) elements which confirmed the successful preparation of nanocatalyst. The EDX mapping in addition to the conventional executed EDAX elemental mapping (1 µm) confirmed a meaningful and uniform distribution of the element in bio-nanocomposite.

**Figure 7:**
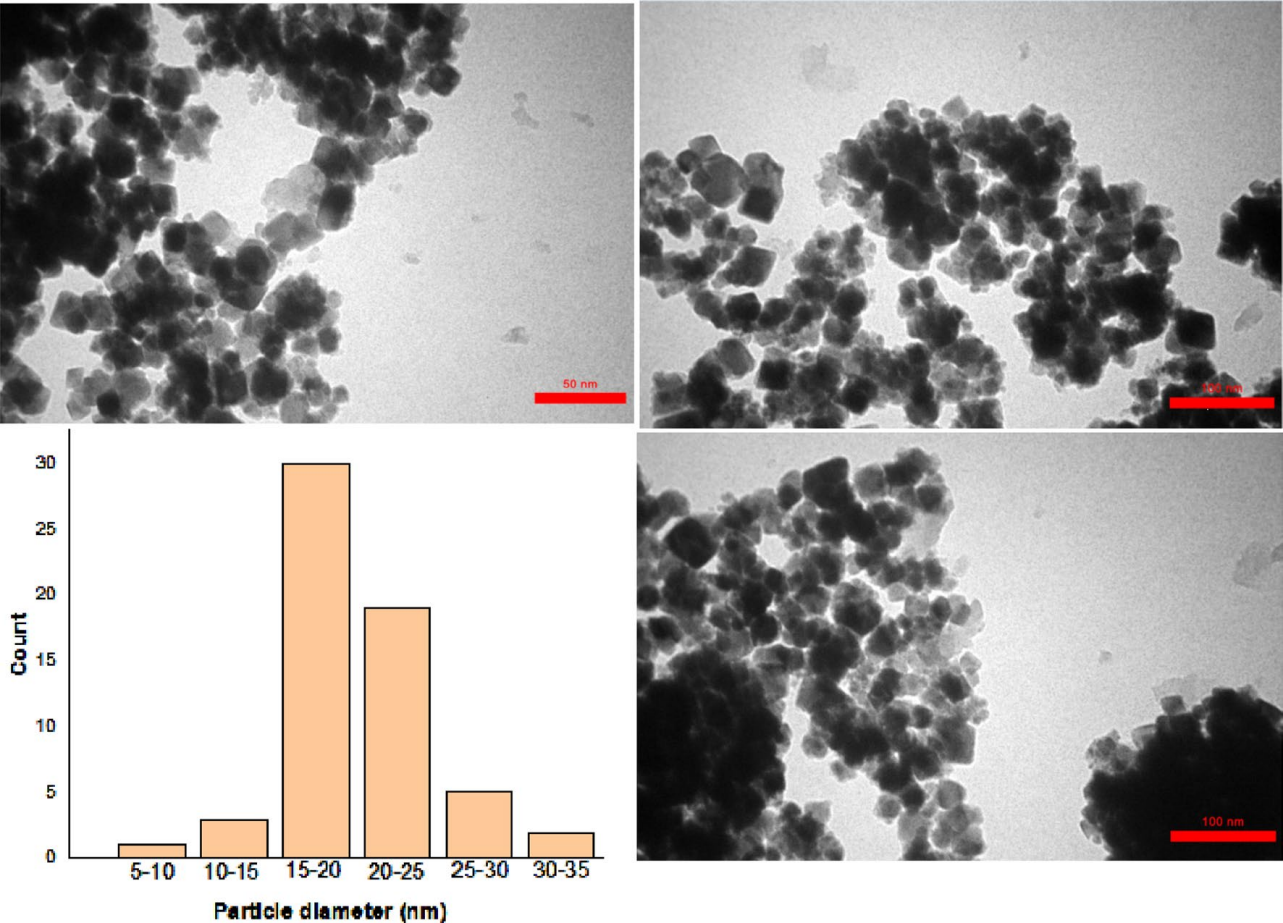
TEM images and particle size histogram distribution of FeAl_2_O_4_-SiO_2_@[*DL*-Ala][NO_3_].

The TEM images (in 100 nm and 50 nm magnifications) and also particle size distribution histogram of the FeAl_2_O_4_-SiO_2_@[*DL*-Ala][NO_3_] bio-nanohybrid presented in Fig. 7. Based on the images, nanocatalyst is made up of various layers that the dark parts related to the internal core while the light moieties around it belongs to sell. The histogram dedicated that the particle size is in a narrow distribution which almost are 15–20 nm.

**Figure 8:**
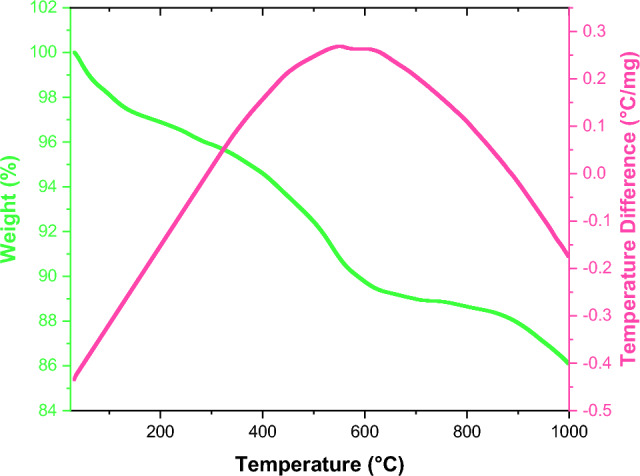
TGA/DSC curves of nano FeAl_2_O_4_-SiO_2_@[*DL*-Ala][NO_3_].

The thermal stability of FeAl_2_O_4_-SiO_2_@[*DL*-Ala][NO_3_] magnetized nanostructure investigated using TGA/DSC diagrams, as shown in Fig. 8. According to diagram, the first weight loss occurred at 558–575 °C (9.41%). Elevation the temperature up to 1000 °C the FeAl_2_O_4_-SiO_2_@[*DL*-Ala][NO_3_] showed the total weight loss of 13.90%. The results affirmed that the catalyst is stable up to 500 °C.

X-ray fluorescence (XRF) analysis of the FeAl_2_O_4_-SiO_2_@[*DL*-Ala][NO_3_] nanostructure illustrated in Table 1. According to the data, presence of Fe_2_O_3_ (76.133%), Al_2_O_3_ (5.505%) and SiO_2_ (11.07%) affirmed successful preparation of nano biocatalyst. It means the nanostructure contains 53.248% Fe, 2.913% Al, and 5.168% Si.

**Table 1:** XRF data of FeAl_2_O_4_-SiO_2_@[*DL*-Ala][NO_3_].

	Fe_2_O_3_	Al_2_O_3_	SiO_2_	L.O.I
%	76.133	5.505	11.07	6.98

**Figure 9:**
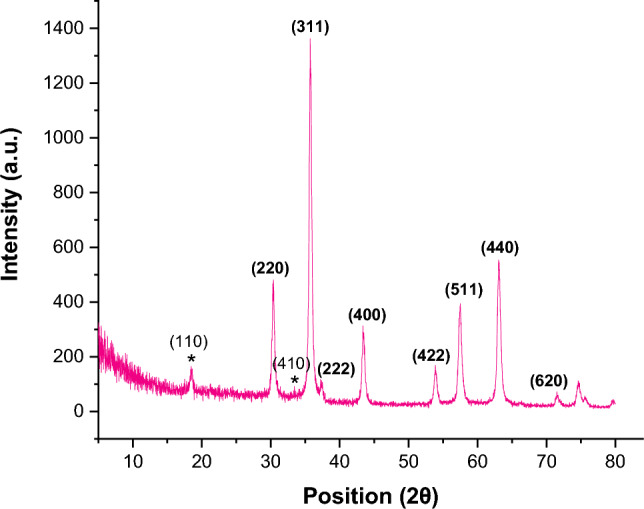
XRD pattern of the FeAl_2_O_4_-SiO_2_@[*DL*-Ala][NO_3_].

The XRD patterns of the FeAl_2_O_4_-SiO_2_@[*DL*-Ala][NO_3_] bio-nanostructure depicted in Fig. 9. The peaks at 2θ = 30.40° (220), 35.77° (311), 37.51° (222), 43.61° (400), 54.01° (422), 57.43° (511), 63.21° (440), and 71.51° (620) identified the crystal structure of FeAl_2_O_4_ core (Reference code: 01–089-1696, ICSD name: Iron Aluminum Oxide). In addition, the peaks at 2θ = 18.39° (110) and 33.43° (410) affirmed the presence of *DL*-Ala in the structure (Reference code: 00–021-1569). It must be mentioned that the *DL*-alanine represented in its cationic form in the [*DL*-Ala][NO_3_] IL which affect its crystal structure. This could be one reason that all of the characteristic peaks of *DL*-alanine didn't observed in the XRD pattern of the final bio-nanostructure. In addition, a broadness centralized at about 22° related to amorphous silica layer.

### Investigation of the catalytic activity of nano FeAl_2_O_4_-SiO_2_@[DL-Ala][NO_3_] in the synthesis of some spiro[chromeno[2,3-c]pyrazole-4,2′-indene]triones and spiro[chromeno[2,3-c]pyrazole-4,3′-indoline]diones

Initially, to investigate the catalytic efficacy of bio-nanocomposite, the reaction of hydrazine hydrate (**1a**) (1 mmol), ethyl acetoacetate (**2**) (1 mmol), ninhydrin (**3a**) (1 mmol), and dimedone (**4a**) (1 mmol) in the presence of FeAl_2_O_4_-SiO_2_@[*DL*-Ala][NO_3_] was chosen as a model reaction to optimize the reaction conditions. According to results in Table 2, different experimental parameters were tested to obtain the optimized situation. The reaction was carried out under solvent-free conditions in different temperatures (entries 1–3). According to the results, although the solvent-free conditions didn't get satisfactory results, but elevating the temperature yielded better reaction promotion. In order to obey the green chemistry rules, the model reaction examined in aqueous media (entries 4–7). The results affirmed that water seems to be good choice. The reaction was performed at 90 oC in different amounts of catalyst (entries 4 and 5). Surprisingly the lower amount of 0.02 g reveled better results. In the next attempt, the reaction examined under aqueous reflux conditions (entries 6 and 7). The best results obtained in the presence of 0.02 g of the nano FeAl_2_O_4_-SiO_2_@[*DL*-Ala][NO_3_] in aqueous reflux conditions (entry 6). Although the main purpose was performing the reaction under aqueous green conditions, but other solvents also examined (entries 8–11) It must be mentioned that all the reactions performed via domino one-pot manner in which an water-mediated mixture of (**1a**), (**2**), and FeAl_2_O_4_-SiO_2_@[*DL*-Ala][NO_3_] bio-nanocomposite stirred at room temperature within 30 min until appearance of 3-methyl-1*H*-pyrazol-5(4*H*)-one intermediate (checked by TLC). Then the substrates (**3a**) and (**4a**) added to the mixture and stirred under reflux conditions for the appropriate time mentioned in Table 2.

**Table 2:** Optimization of reaction conditions for the synthesis of 3,7,7-trimethyl-7,8-dihydro-1*H*-spiro[chromeno[2,3-*c*]pyrazole-4,2′-indene]-1′,3′,5(6*H*)-trione (**5a**).

Entry	Nano catalyst (g)	Solvent (3 mL)	Temperature (°C)	Time (h)^b^	Yield (%)
1	0.04	Solvent-free	rt	15	˂ 5
2	0.04	Solvent-free	70	15	30
3	0.04	Solvent-free	90	15	40
4	0.04	H_2_O	90	10	60
5	0.02	H_2_O	90	8	70
6	0.02	H_2_O	Reflux	8	80
7	0.024	H_2_O	Reflux	8	80
8	0.02	MeOH	Reflux	8	60^a^
9	0.02	EtOH	Reflux	8	65
10	0.02	MeOH/H_2_O (1:1)	Reflux	8	70
11	0.02	EtOH/H_2_O (1:1)	Reflux	8	65

^a^Some by-products observed.

^b^The time refers to maximum reaction progress and further time-elongation hasn't led to more progression.

In order to evaluate the efficacy of the bio-based multi-layered nanostructure (nano FeAl_2_O_4_-SiO_2_@[*DL*-Ala][NO_3_]) to promote the model reaction, the synthesis of (**5a**) examined under catalyst-free conditions as well as the presence of each layer of the catalyst. All the entries proceeded via a domino manner in which a mixture of hydrazine hydrate (**1a**) and ethyl acetoacetate (**2**), in 1:1 molar ratio, in the presence of the mentioned catalyst mixed in water (3 mL) for 30 min. Then equimolar amounts of ninhydrin (**3a**) and dimedone (**4a**) added and refluxed for the mentioned times in the Table 3. According to the results summarized in Table 3, the crucial role of the nanostructure is clear.

**Table 3:** Screening the catalyst efficacy for the synthesis of (**5a**).

Entry	Nano catalyst (0.02 g)	Time (h)^b^	Yield (%)
1	–	10	–
2	FeAl_2_O_4_	9	50
3	FeAl_2_O_4_-SiO_2_	9	60
4	[*DL*-Ala][NO_3_]	8	50
5	FeAl_2_O_4_-SiO_2_@[*DL*-Ala][NO_3_]	8	80

Based on the optimized reaction conditions, different spiro[chromeno[2,3-*c*]pyrazole-4,2′-indene]triones and spiro[chromeno[2,3-*c*]pyrazole-4,3′-indoline]diones prepared successfully via a domino one-pot situations. The results are summarized in Table 4. The reaction of different hydrazines (hydrazine hydrate, phenylhydrazine, 4-nitrophenylhydrazine, and 2,4-dinitrophenyl hydrazine) with ethyl acetoacetate, ninhydrin, and dimedone occurred successfully to obtain the corresponding **5a–c** products. The reaction of hydrazines (hydrazine hydrate and phenylhydrazine) with ethyl acetoacetate, ninhydrin, and 1,3-cyclohexanedione occurred successfully to obtain the corresponding (**5d–f**) products. In order to obtain diverse spiroheterocycles, isatin utilized, as a heterocyclic 1,2-diketone candidate, which resulted to good results (**5g-h**). In order to vast the method scope, 5-bromoisatin also yielded successful results with different hydrazines and 1,3-diketones (dimedone and 1,3-cyclohexanedione) that gained the products **5i-n** successfully. The known compounds characterized though the comparison of their data with the corresponding authentic samples^33^. The **5n** are new compound which specified completely by physical and spectral data. Baes on the data of Table 3, it seems utilizing isatin and 5-bromoisatin instead of ninhydrin yielded the corresponding adducts in shorter reaction times, which could be due to more sterically-hindered structure of ninhydrin in comparison to isatins.

**Table 4:**
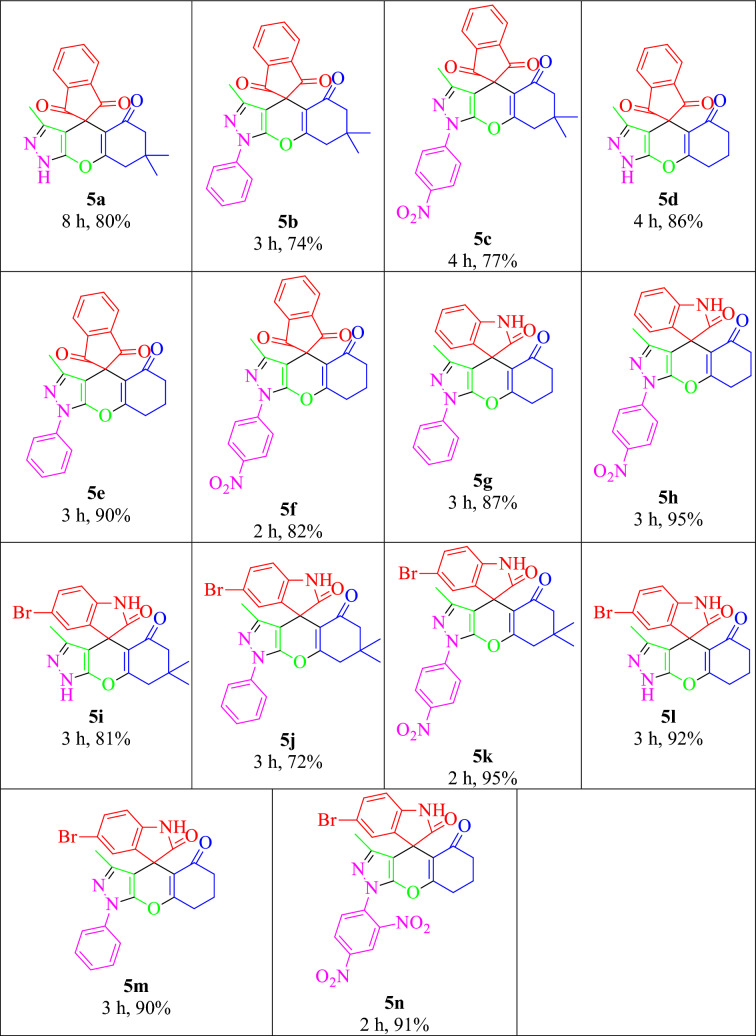
Synthesis of dihydro-1*H*-spiro[chromeno[2,3-*c*]pyrazole-4,2′-indene]-1′,3′,5(6*H*)-triones and dihydro-1*H*-spiro[chromeno[2,3-*c*]pyrazole-4,3′-indoline]-2′,5(6*H*)-diones^a^.

^a^Reaction conditions: a mixture of hydrazines (**1a-d**) and ethyl acetoacetate (**2**), in 1:1 molar ratio, in the presence of FeAl_2_O_4_-SiO_2_@[*DL*-Ala][NO_3_] (0.02 g) mixed in water (3 mL) for 30 min. Then equimolar amounts of cyclic diketones (**3a-c**) and 1,3-diketones (**4a-b**) added and refluxed for the mentioned times in the Table.

**Figure 10:**
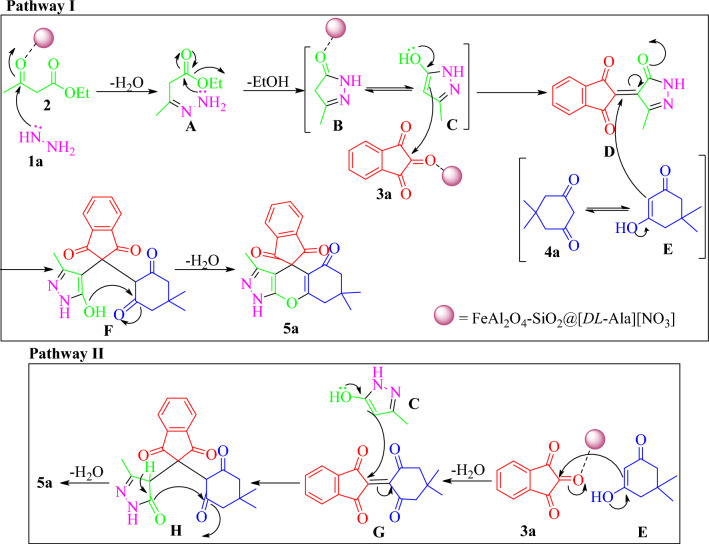
Scheme of the proposed mechanism for the synthesis of 3,7,7-trimethyl-7,8-dihydro-1*H*-spiro[chromeno[2,3-*c*]pyrazole-4,2′-indene]-1′,3′,5(6*H*)-trione (5a).

Although the reaction route is not clear completely, the plausible reaction mechanism for the formation of (**5a**) illustrated in Fig. 10 according to the previously reported observation^33^. The reaction could proceed via two pathways. In pathway (**I**), the pyrazolone (**B**) is produced via the reaction of hydrazine hydrate (**1a**) with ethyl acetoacetate (**2**) in presence of nano FeAl_2_O_4_-SiO_2_@[*DL*-Ala][NO_3_] in water. Compound (**B**) undergoes the tautomeric equilibrium with its enol form (**C**). The Knoevenagel condensation of (**C**) with ninhydrin (**3a**) yielded the intermediate (**D**). The tautomeric form of dimedone (**E**) performed the 1,4-nucleophilic addition with *α,β*-unsaturated ketone (**D**) led to intermediate (**F**), which underwent intramolecular dehydrative cyclization to form the desired product (**5a**). On the other hand, the product (**5a**), also could gained form the pathway (**II**), in which the enolic tautomer of dimedone (**E**) done the dehydrative nucleophilic attack to ninhydrin (**3a**) to obtain (**G**). The subsequent Michael addition of (**G**) with intermediate (**C**) generate (**H**). Finally, compound (**5a**) is obtained via the dehydrative cyclization route of compound (**H**). Totally, the ionic characteristic of the [*DL*-Ala][NO_3_] IL in the outer layer of the bio-nanocomposite, in addition to the acidic properties of the FeAl_2_O_4_ core and silica middle layer, causes the activation of different functional groups in the substrates/intermediates. Actually, the bio-nanohybrid FeAl_2_O_4_-SiO_2_@[*DL*-Ala][NO_3_], possess high catalytic efficacy due to synergic effects of intrinsic promotion characteristics of each of its layers.

**Figure 11:**
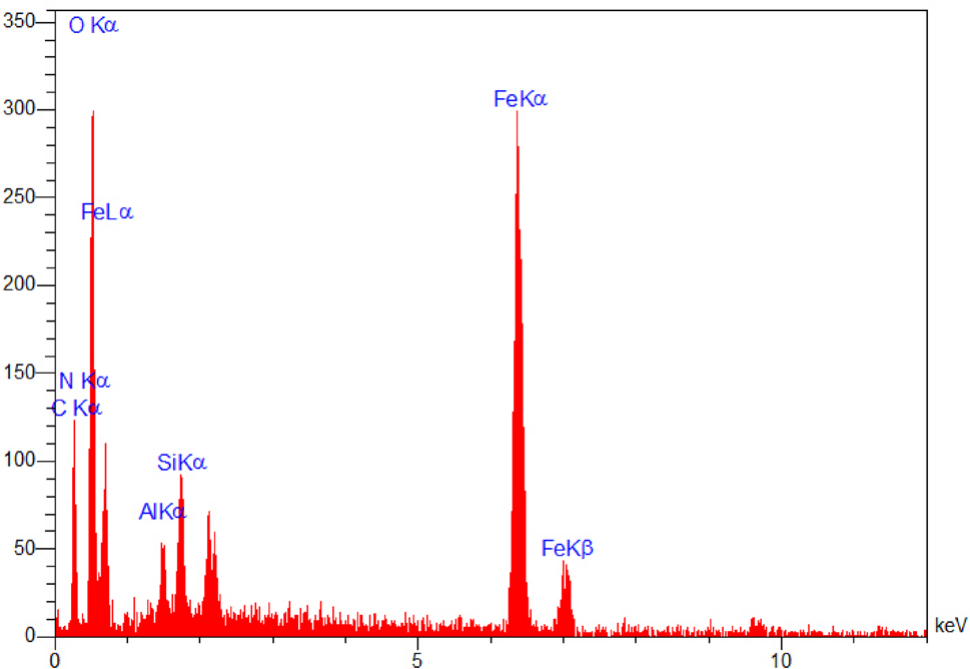
The EDAX analysis of the reused FeAl_2_O_4_-SiO_2_@[*DL*-Ala][NO_3_] after two runs.

**Figure 12:**
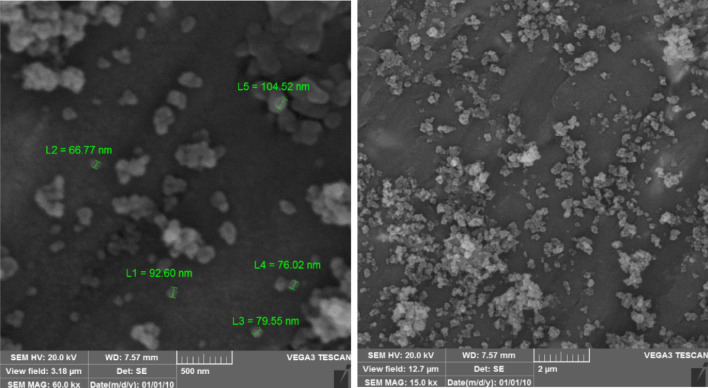
FESEM images of the recovered FeAl_2_O_4_-SiO_2_@[*DL*-Ala][NO_3_].

In the next step, the recovery and reusability of FeAl_2_O_4_-SiO_2_@[*DL*-Ala][NO_3_] nanostructure examined for the synthesis of 5′-bromo-1-(2,4-dinitrophenyl)-3-methyl-7,8-dihydro-1*H*-spiro[chromeno[2,3-*c*]pyrazole-4,3′-indoline]-2′,5(6*H*)-dione (**5n**) through the one-pot domino aqua-mediated reaction of equimolar amounts of 2,4-dinitrophenyl hydrazine (**1d**), ethyl acetoacetate (**2**), 5-bromoisatin (**3c**), and 1,3-cyclohexanedione (**4b**) in the presence of FeAl_2_O_4_-SiO_2_@[*DL*-Ala][NO_3_] (0.02 g). After completion of the reaction (2 h), distilled water (25 mL) added to the cooled mixture and the catalyst was separated with an external magnet. Subsequent washing of the recovered FeAl_2_O_4_-SiO_2_@[*DL*-Ala][NO_3_] by distilled water (3 × 10 mL), followed by air-drying for 2 h and oven-drying at 75 °C for 5 h, ultimate the recovered bio-nanostructure to be utilized for another cycle to prepare (**5n**), which performed the reaction successfully by 90% within 2 h. The EDAX data of the reused FeAl_2_O_4_-SiO_2_@[*DL*-Ala][NO_3_] bio-nanostructure illustrated in Fig. 11. The elements: Fe (80.78 W%), Al (0.70 W%), O (8.08 W%), Si (1.21 W%), C (8.51 W%) and N (0.72 W%) observed in the recycles bio-nanostructure. The FESEM images of the reused and recovered FeAl_2_O_4_-SiO_2_@[*DL*-Ala][NO_3_] bio-nanohybrid (in 2 µm and 500 nm magnetization) are demonstrated in Fig. 12. The average size of nanoparticles is between 75–100 nm and the morphology and particles size remained invariable after two runs, except some agglomeration occurred in comparison to the initial nanostructure (Fig. 5).

Finally in order to compare the efficacy of FeAl_2_O_4_-SiO_2_@[*DL*-Ala][NO_3_] inorganic-bioorganic nanohybrid with previously reported promotors, the preparation of 3-methyl-1-(4-nitrophenyl)-7,8-dihydro-1*H*-spiro[chromeno[2,3-*c*]pyrazole-4,3′-indoline]-2′,5(6*H*)-dione (**5 h**), via a domino four-component reaction, illustrated in Table 5.

**Table 5:** Comparing preparation of **5h** with the previously reported procedure.

Entry	Conditions	Time (h)/yield (%)	Reference
1	Dodecylbenzenesulphonic acid (DBSA) (10 mol%)/H_2_O (3 mL)/90 °C	8/82	^33^
2	Nano FeAl_2_O_4_-SiO_2_@[*DL*-Ala][NO_3_] (0.02 g)/H_2_O (3 mL)/reflux	3/95	This work

## Experimental

### General

All reagents and solvents purchased from the Merck, Aldrich and Alfa-Aesar companies and used without any additional purification. Melting points were measured using an electrothermal 9200 apparatus and reported uncorrected. FT-IR spectra of catalyst and samples were taken using a Bruker FT-IR (tensor 27) spectrometer with potassium bromide pellets. Magnetization properties were evaluated by VSM (MDKB) apparatus. The morphology, shape and size of the nanocatalyst was observed via FESEM (VEGA\\TESCAN-LMU and mira). Element percentages were estimated with Philips-PW2404 XRF spectrometer. TEM image was taken by a CM120 electron microscope. TGA analysis was recorded using a TA Q600 thermogravimetric analyzer. The EDAX data and elemental mapping were analyzed by a VEGA3 TESCAN and MIRA TESCAN apparatus. The XRD pattern of the nanostructure was carried out by a X′ Pert MPD. Homogenization of the particles was done in a Wise clean (power of 90 W) ultrasonic bath. A UNIVERSAL 320 centrifuge apparatus (5000–10,000 rpm) was applied for the preparation of the bionanohybride. ^1^H NMR and ^13^C NMR spectra were performed using a Bruker Advance-III 300 MHz spectrometer in DMSO-*d*_*6*_ as solvent. The sample mass was identified by Mass device (MS (5973) Agilent Technologies).

### ***Preparation of FeAl***_***2***_***O***_***4***_***-SiO***_***2***_***@[DL-Ala][NO***_***3***_***] MNPs***

#### *Preparation of nano FeAl*_*2*_*O*_*4*_

Synthesis of nano FeAl_2_O_4_ was done by a chemical co-precipitation technique reported previously with some modifications^48^. First, a mixture of Al(NO_3_)_3_.9H_2_O (0.375 g, 1 mmol) and FeCl_2_.4H_2_O (0.795 g, 4 mmol) dissolved in distilled water (100 mL) under N_2_ atmosphere at 80 °C within 30 min. Next, NaOH solution (2 M, 6 mL) was added dropwise into the stirring mixture for 10 min until the pH reached 12 that checked with pH paper. The mixture stirred at 80 °C under N_2_ atmosphere for 30 min. The solid was separated using an external magnet, washed with deionized water (3 × mL). The obtained solid dried at 75 °C overnight to get a black powder.

#### *Preparation of FeAl*_*2*_*O*_*4*_*-SiO*_*2*_

The process was accomplished through a modified procedure^46^. In a flask, a mixture of FeAl_2_O_4_ nanoparticles (1 g) were dispersed in a mixture of ammonia (25 wt %, 2 mL), distilled water (20 mL), and absolute ethanol (60 mL) sonicated in a sonic-bath for 30 min. In the next step, a solution consists of tetraethyl orthosilicate (TEOS, 0.5 mL) in absolute ethanol (1 mL) was added dropwise into the FeAl_2_O_4_ nanoparticle solution under vigorous stirring at room temperature. The mixture was then stirred for 20 h at room temperature. Finally, the products were separated by an external magnet, and washed with absolute ethanol (3 × 5 mL). The solid was dried at 70 °C for 5 h to yield FeAl_2_O_4_-SiO_2_ as a black powder.

#### *Preparation of [DL-Ala][NO*_*3*_*] ionic liquid*

The [*DL*-Ala][NO_3_] ionic liquid was prepared based on a previously reported procedure^51^ with some modifications Typically, aqueous mixture of HNO_3_ (1 M, 20 mL) and *DL*-alanine (1 M, 20 mL) stirred magnetically at 60 °C at 500 rpm for 5 h, followed by oven-drying at 95 °C for 3 h. The [*DL*-Ala][NO_3_] IL obtained as a black viscous liquid. IR (KBr, cm^−1^): 3061, 1740, 1648, 1517, 1349, 1207, 1117, 1041, 916, 770, 529. ^1^H-NMR (300 MHz, DMSO-*d*_*6*_): 1.38 (d, 3H, *J* = 6.6 Hz, CH_3_), 3.89–3.92 (m, 1H, CH), 7.11–7.81 (bs, 3H, NH_3_^+^), 8.26 (bs, OH) ppm. ^13^C-NMR (75 MHz, DMSO-*d*_*6*_): 16.21, 48.55, 172.07 ppm.

#### *Preparation of FeAl*_*2*_*O*_*4*_*-SiO*_*2*_*@[DL-Ala][NO*_*3*_*] MNPs*

FeAl_2_O_4_-SiO_2_ nanoparticles (1 g) and [*DL*-Ala][NO_3_] ionic liquid (1 g) was dispersed in distilled water (30 mL) and stirred for 24 h at room temperature. Then, the solid were collected by an external magnet and washed by distilled water (3 × 10 mL). The obtained precipitate dried at room temperature for 2 h and next dried at 75 °C for 7 h to get the multi-layered bio-nanostructure as a black solid.

#### *General procedure for the synthesis of tricyclic dihydro-spiro[chromeno[2,3-c]pyrazole-4,2*′*-indene]triones and dihydro-spiro[chromeno[2,3-c]pyrazole-4,3*′*-indoline]diones (5a-n)*

To a solution of hydrazines (**1a–d**) (1 mmol) and ethyl acetoacetate (**2**) (1 mmol) in water (3 mL), the nano FeAl_2_O_4_-SiO_2_@[*DL*-Ala][NO_3_] (0.02 g) was added and stirred magnetically for 30 min at room temperature. Next, cyclic diketones (**3a–c**) (1 mmol) and cyclic 1,3-dicarbonyls (**4a, b**) (1 mmol) added to the reaction mixture and stirred under reflux conditions for the appropriate time monitored by TLC (*n*-hexane/EtOAc 1:1). After completion of the reaction, the mixture was cooled. Distilled water (25 mL) added to the mixture and the catalyst was separated with an external magnet. The crude solution extracted with ethyl acetate (50 mL). Purification of the product was accomplished through thin-layer chromatography (PLC).

5′-Bromo-1-(2,4-dinitrophenyl)-3-methyl-7,8-dihydro-1H-spiro[chromeno[2,3-c]pyrazole-4,3′-indoline]-2′,5(6H)-dione (**5n**). Brown powder; m.p. = 80 °C, IR (KBr): 3216, 2946, 1716, 1670, 1616, 1522, 1386, 1338, 1220, 1186, 1132, 984, 816, 670, 539, 455, 420. ^1^H NMR (300 MHz, DMSO): 1.88 (bs, 2H, CH_2_), 2.23 (s, 3H, CH_3_), 2.37 (bs, 2H, CH_2_), 2.67 (bs, 2H, CH_2_), 6.68–6.88 (m, 2H, Ar), 7.08 (s, 1H, Ar), 7.19–7.24 (m, 2H, Ar), 7.55 (s, 1H, Ar), 10.48 (bs, 1H, NH). ^13^C NMR (75 MHz, DMSO): 20.11, 20.43, 20.62, 27.39, 33.20, 35.60, 37.56, 46.02, 47.27, 110.57, 111.53, 112.52, 114.01, 114.57, 125.95, 128.11, 130.80, 137.09, 143.76, 158.58, 166.09, 178.51, 195.64, 195.87. MS (ESI) m/z 566 [M]^+^, 551 ([M]^+^–Me), 523 ([M]^+^–NHCO], 495 ([M]^+^–NHCO, –C_2_H_4_), 466 ([M]^+^–NHCO, –CO, –C_2_H_4_), 413 (M]^+^–C_6_H_4_Br), 357 ([M]^+^–(NO_2_)_2_C_6_H_3_, –CH_3_, –N_2_), 303 ([M]^+^–(NO_2_)_2_C_6_H_3_, –CH_3_, –Br), 279 ([M]^+^-cyclohexanone, –Me, –2NO_2_, –OH, –Br), 166 [(NO_2_)_2_C_6_H_3_]^+^, 98 [cyclohexanone]^+^, 77 [C_6_H_5_]^+^.

## Conclusion

In this research, a multi-layered magnetic bio-nanostructure based on *DL*-alanine amino acid (FeAl_2_O_4_-SiO_2_@[*DL*-Ala][NO_3_]) prepared (with the average particle size of 55–95 nm) via step-by-step simple method from spinel FeAl_2_O_4_ core and identified by FT-IR, VSM, FESEM, EDAX, TEM, TGA/DSC, XRF, and XRD techniques. The catalytic performance of the bio-nanocomposite investigated for the synthesis of two classes of heterocycles via domino four-component reactions in green aqueous media. Dihydro-1*H*-spiro[chromeno[2,3-*c*]pyrazole-4,2′-indene]-1′,3′,5(6*H*)-triones and dihydro-1*H*-spiro[chromeno[2,3-*c*]pyrazole-4,3′-indoline]-2′,5(6*H*)-diones obtained from hydrazine derivatives, ethyl acetoacetate, cyclic ketones and cyclic 1,3-diketones successfully by 72–95%. The highlighted features of this procedure are high yields of the products, short reaction times, simple work-up procedure and green conditions in the absence of hazardous solvent/additives, and easy separation of the bio-promotor (through an external magnet) and its efficient reusability. In addition, the procedure yielded preparation of some interesting classes of heterocyclic systems in the presence of a novel FeAl_2_O_4_-SiO_2_@[*DL*-Ala][NO_3_] inorganic-bioorganic nanohybrid, successfully.

### Supplementary Information

Supplementary Information.

## Data Availability

The datasets used and/or analyzed during the current study are available from the corresponding author on reasonable request.
