# Supplementary material for: Nano silicated-FeAl2O4 functionalized by DL-alaninium nitrate ionic liquid (FeAl2O4-SiO2@[DL-Ala][NO3]) as versatile promotor for aqua-mediated synthesis of spiro[chromenopyrazole-indene-triones and spiro[chromenopyrazole-indoline-diones
Source: Sci Rep. 2024 Jul 15;14:16296. doi: 10.1038/s41598-024-66750-2 (PMC11251080; doi:10.1038/s41598-024-66750-2)
Supplement: Supplementary file 1 — Supplementary Information. [file 41598_2024_66750_MOESM1_ESM.docx]

**Nano silicated-FeAl_2_O_4_ functionalized by *DL*-alaninium nitrate ionic liquid (FeAl_2_O_4_-SiO_2_@[*DL*-Ala][NO_3_]) as versatile promotor for aqua-mediated synthesis of** **spiro[chromenopyrazole-indene-triones and spiro[chromenopyrazole-indoline-diones**


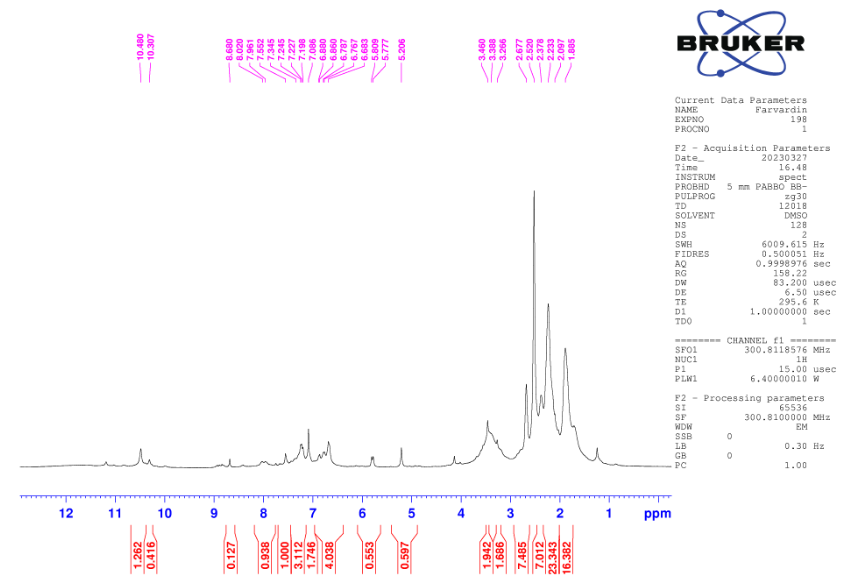


**SI-1** ^1^H-NMR (300 MHz, DMSO-*d_6_*) of 5'-bromo-1-(2,4-dinitrophenyl)-3-methyl-7,8-dihydro-1H-spiro[chromeno[2,3-c]pyrazole-4,3'-indoline]-2',5(6H)-dione (**5n**).


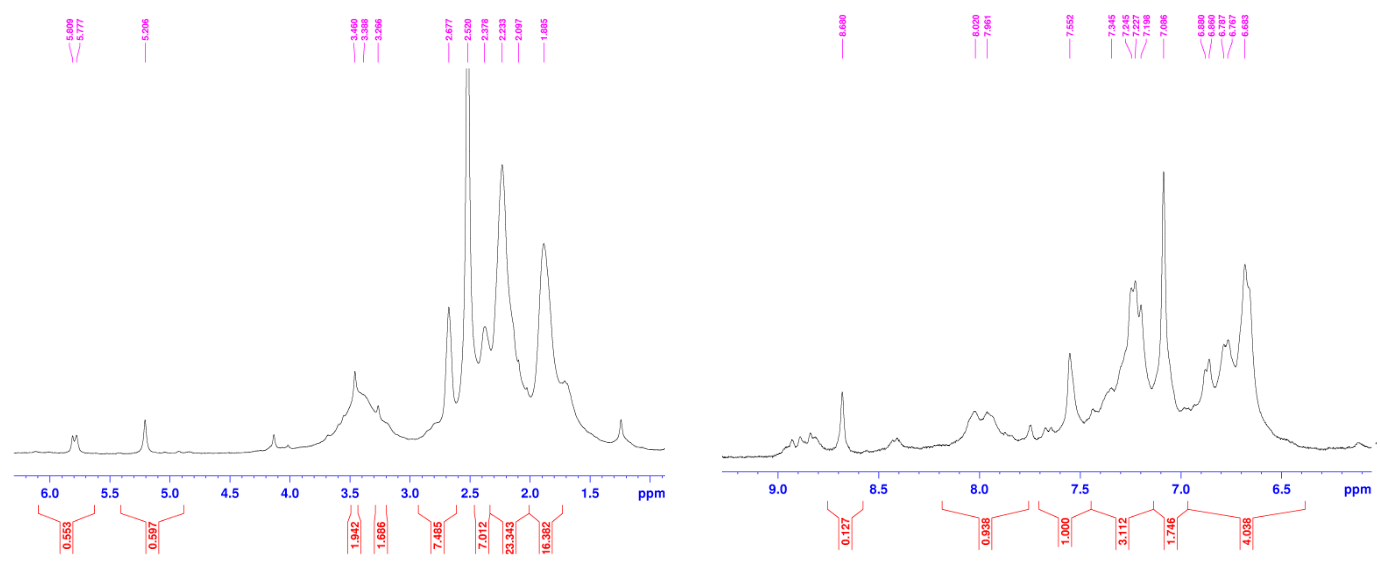


**SI-2** Expanded ^1^H-NMR (300 MHz, DMSO-*d_6_*) of 5'-bromo-1-(2,4-dinitrophenyl)-3-methyl-7,8-dihydro-1H-spiro[chromeno[2,3-c]pyrazole-4,3'-indoline]-2',5(6H)-dione (**5n**).


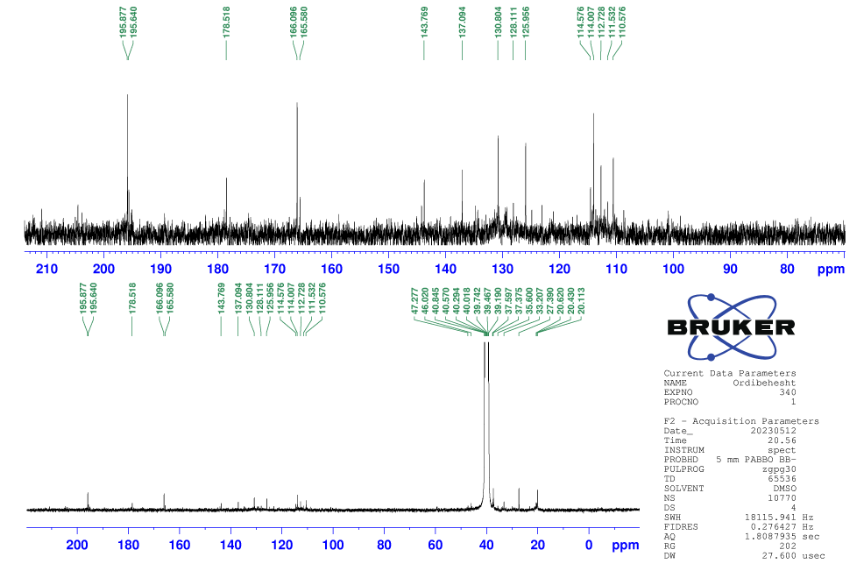


**SI-3** ^13^C-NMR (75 MHz, DMSO-*d_6_*) of 5'-bromo-1-(2,4-dinitrophenyl)-3-methyl-7,8-dihydro-1H-spiro[chromeno[2,3-c]pyrazole-4,3'-indoline]-2',5(6H)-dione (**5n**).


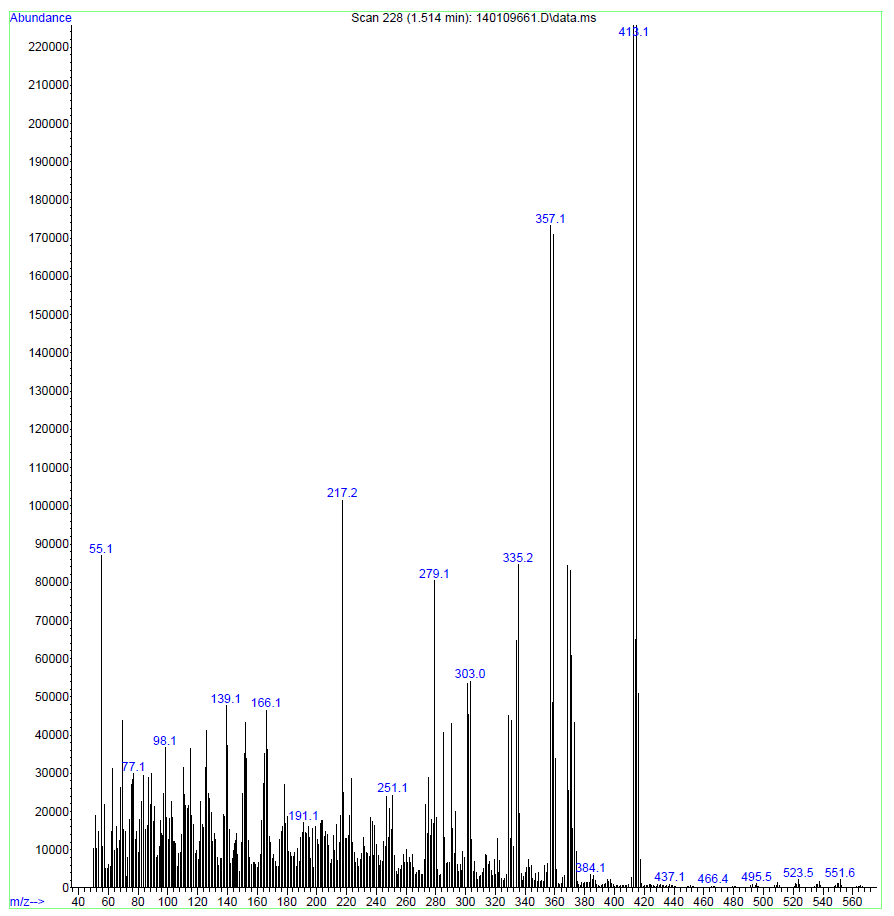


**SI-4** MASS spectra of 5'-bromo-1-(2,4-dinitrophenyl)-3-methyl-7,8-dihydro-1H-spiro[chromeno[2,3-c]pyrazole-4,3'-indoline]-2',5(6H)-dione (**5n**).


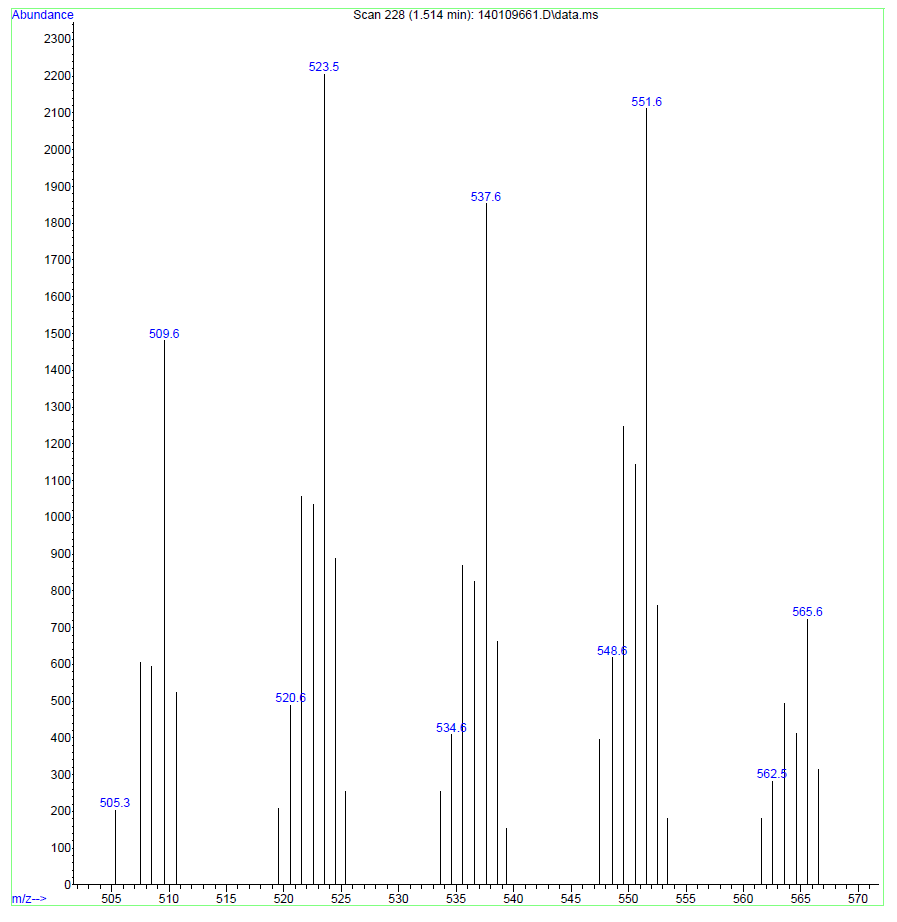


**SI-5** Expanded MASS spectra of 5'-bromo-1-(2,4-dinitrophenyl)-3-methyl-7,8-dihydro-1H-spiro[chromeno[2,3-c]pyrazole-4,3'-indoline]-2',5(6H)-dione (**5n**).


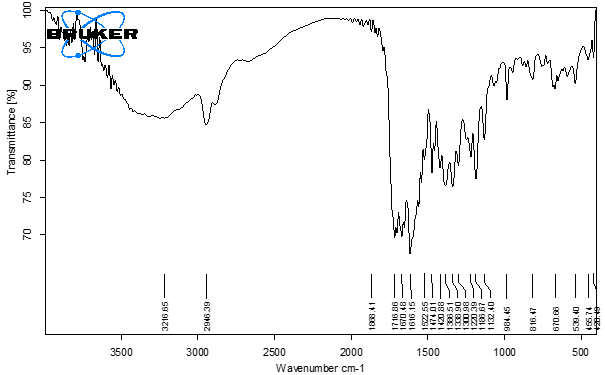


**SI-6** FT-IR spectra of 5'-bromo-1-(2,4-dinitrophenyl)-3-methyl-7,8-dihydro-1H-spiro[chromeno[2,3-c]pyrazole-4,3'-indoline]-2',5(6H)-dione (**5n**).


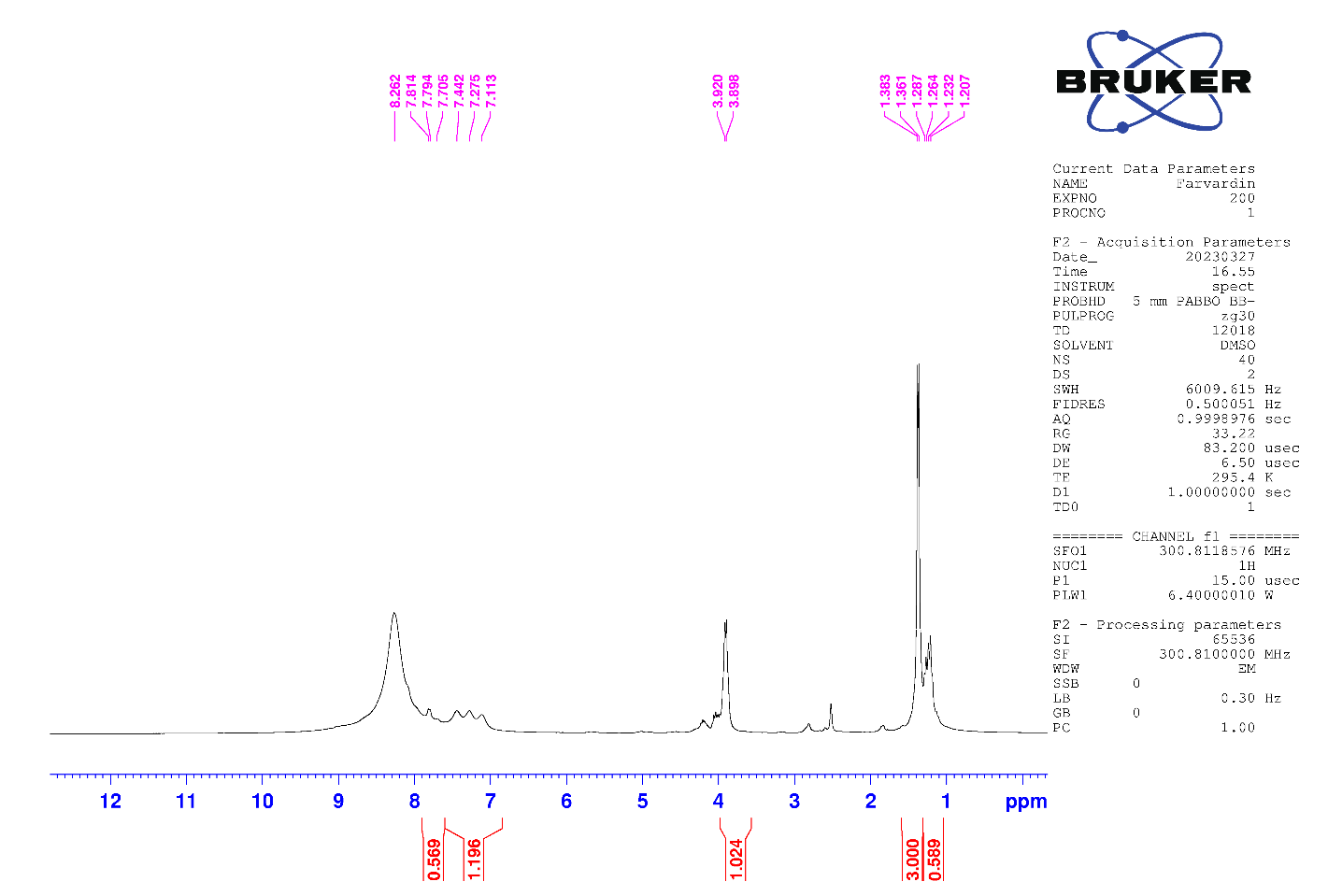


**SI-7** ^1^H-NMR (300 MHz, DMSO-*d_6_*) of [*DL*-Ala][NO_3_] ionic liquid.


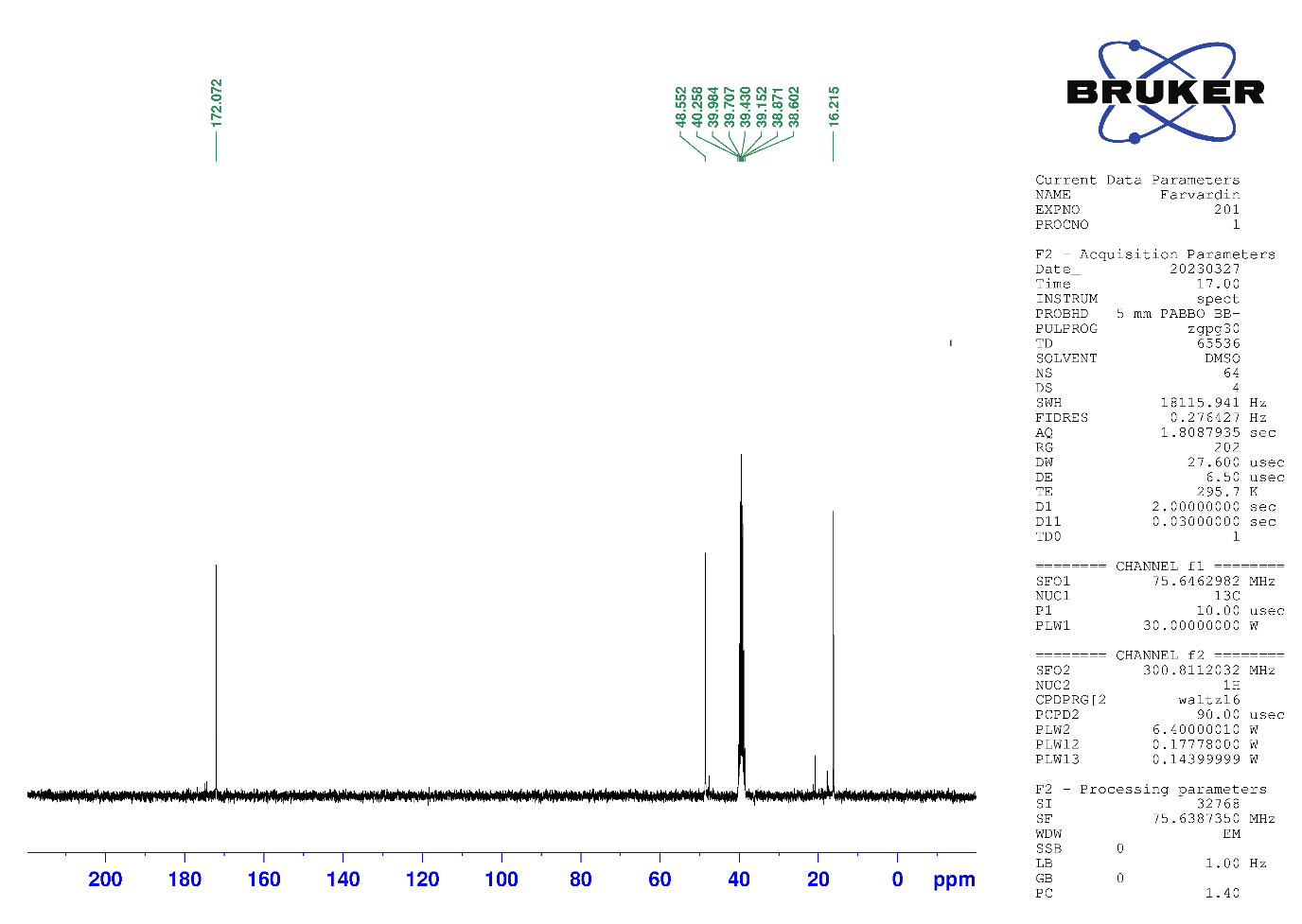


**SI-8** ^13^C-NMR (75 MHz, DMSO-*d_6_*) of [*DL*-Ala][NO_3_] ionic liquid.


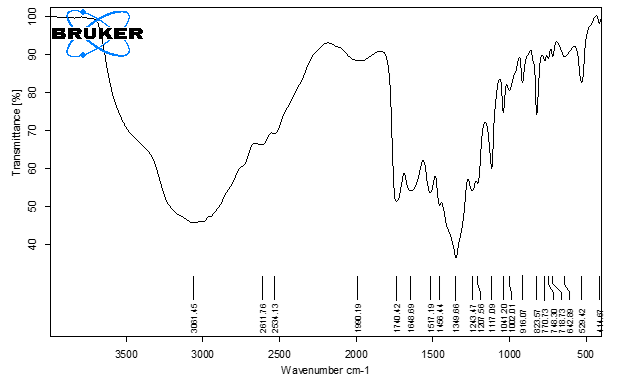


**SI-9** FT-IR spectra of [*DL*-Ala][NO_3_] ionic liquid.
